# Pre-Clinical Assays Predict Pan-African *Echis* Viper Efficacy for a Species-Specific Antivenom

**DOI:** 10.1371/journal.pntd.0000851

**Published:** 2010-10-26

**Authors:** Nicholas R. Casewell, Darren A. N. Cook, Simon C. Wagstaff, Abdulsalami Nasidi, Nandul Durfa, Wolfgang Wüster, Robert A. Harrison

**Affiliations:** 1 Alistair Reid Venom Research Unit, Liverpool School of Tropical Medicine, Liverpool, United Kingdom; 2 School of Biological Sciences, Bangor University, Environment Centre Wales, Bangor, United Kingdom; 3 Federal Ministry of Health, Abuja, Nigeria; University of Melbourne, Australia

## Abstract

**Background:**

Snakebite is a significant cause of death and disability in subsistent farming populations of sub-Saharan Africa. Antivenom is the most effective treatment of envenoming and is manufactured from IgG of venom-immunised horses/sheep but, because of complex fiscal reasons, there is a paucity of antivenom in sub-Saharan Africa. To address the plight of thousands of snakebite victims in savannah Nigeria, the EchiTAb Study Group organised the production, testing and delivery of antivenoms designed to treat envenoming by the most medically-important snakes in the region. The *Echis* saw-scaled vipers have a wide African distribution and medical importance. In an effort to maximise the clinical utility of scarce antivenom resources in Africa, we aimed to ascertain, at the pre-clinical level, to what extent the *E. ocellatus*-specific EchiTAbG antivenom, which was designed specifically for Nigeria, neutralised the lethal activity of venom from two other African species, *E. pyramidum leakeyi* and *E. coloratus*.

**Methodology/Principal Findings:**

Despite apparently quite distinctive venom protein profiles, we observed extensive cross-species similarity in the immuno-reactivity profiles of *Echis* species-specific antisera. Using WHO standard pre-clinical *in vivo* tests, we determined that the monospecific EchiTAbG antivenom was as effective at neutralising the venom-induced lethal effects of *E. pyramidum leakeyi* and *E. coloratus* as it was against *E. ocellatus* venom. Under the restricted conditions of this assay, the antivenom was ineffective against the lethal effects of venom from the non-African *Echis* species, *E. carinatus sochureki*.

**Conclusions/Significance:**

Using WHO-recommended pre-clinical tests we have demonstrated that the new anti-*E. ocellatus* monospecific antivenom EchiTAbG, developed in response to the considerable snakebite-induced mortality and morbidity in Nigeria, neutralised the lethal effects of venoms from *Echis* species representing each taxonomic group of this genus in Africa. This suggests that this monospecific antivenom has potential to treat envenoming by most, perhaps all, African *Echis* species.

## Author Summary

Snakebite is principally a health concern of rural poor communities. The high snakebite risk of subsistence farming and paucity of effective antivenoms in sub-Saharan Africa means that many communities remain unacceptably vulnerable to snakebite mortality and morbidity. There is therefore a compelling need to maximise the utility of the snakebite therapies that are available. To address Nigeria's severe snakebite problem, the government funded a collaboration of ministry officials, antivenom manufacturers and academics (the EchiTAb Study Group) to produce, test and deliver antivenom. Accordingly, we prepared EchiTAbG, an antivenom specific for envenoming by the saw-scaled viper (*E. ocellatus*) which is responsible for 80% of snakebite deaths in Nigeria. Since *E. ocellatus* is widely distributed across the West African savannah, EchiTAbG offers considerable therapeutic promise in many countries in the region. Since other *Echis* species represent public health concerns elsewhere in Africa, the objective of this study was to examine the pre-clinical intra-generic venom-neutralising efficacy of EchiTAbG. Our results suggest that EchiTAbG (Nigeria registration: A6-0078) has pan-African efficacy against *Echis* envenoming indicating that costly investment in region-specific antivenoms therefore may not be required. This represents an important progression to minimise development costs and maximise the delivery of snakebite therapy for the continent.

## Introduction

The rural communities of sub-Saharan Africa suffer the multiple burdens of low economic status, inadequate access to effective health care and the debilitating effects of numerous infectious and parasitic diseases. The subsistence agriculture livelihood, non-mechanised farming techniques, remote locations and proximity of homes to farms/grain stores all contribute to the fact that these communities also suffer a disproportionally high snakebite mortality rate [1]. Extrapolations from recent global snakebite incidence and mortality data [2] reveal that while the percent lethality of snakebite in Latin America is 1.8% (2,300 deaths; 129,000 incidences) it is 7.6% in sub-Saharan Africa (32,000 deaths; 420,000 incidences). These rather crude data-extrapolations are presented simply to emphasise the point that circumstances in sub-Saharan Africa make snakebite a more life-threatening event than elsewhere.

While socioeconomic issues at the community level and per capita government expenditure on health at the national level certainly contribute to this disparity [1], an important additional explanation is the relative scarcity of antivenom in Africa [3]–[6]. IgG antivenom is the most effective treatment of systemic snake envenoming. However, its manufacture from sera of venom-immunised horses or sheep means that antivenom is a more expensive therapy (US$100/vial in S Africa) than many other non-subsidised medicines administered in sub-Saharan Africa. As described in the cited and related literature, the (i) relatively high cost of antivenom, (ii) its restricted efficacy to the species of snake whose venom was used in its manufacture and (iii) factors relating to commercial manufacturing incentives have all combined to severely limit the availability of antivenom in Africa. There is therefore a compelling need to maximise the clinical utility of effective antivenoms that are becoming available in the region.

In response to the crisis in antivenom supply affecting Nigeria, the EchiTAb Study Group (a collaboration between the Nigerian Federal Ministry of Health, antivenom manufacturers in Costa Rica (Instituto Clodomiro Picado) and Wales (MicroPharm Ltd) and academics in the Liverpool School of Tropical Medicine and University of Oxford) has organised the production, pre-clinical testing [7], [8], human efficacy testing [9] and delivery of two new antivenoms for Nigeria, EchiTAb-Plus-ICP and EchiTAbG.

**Figure 1:**
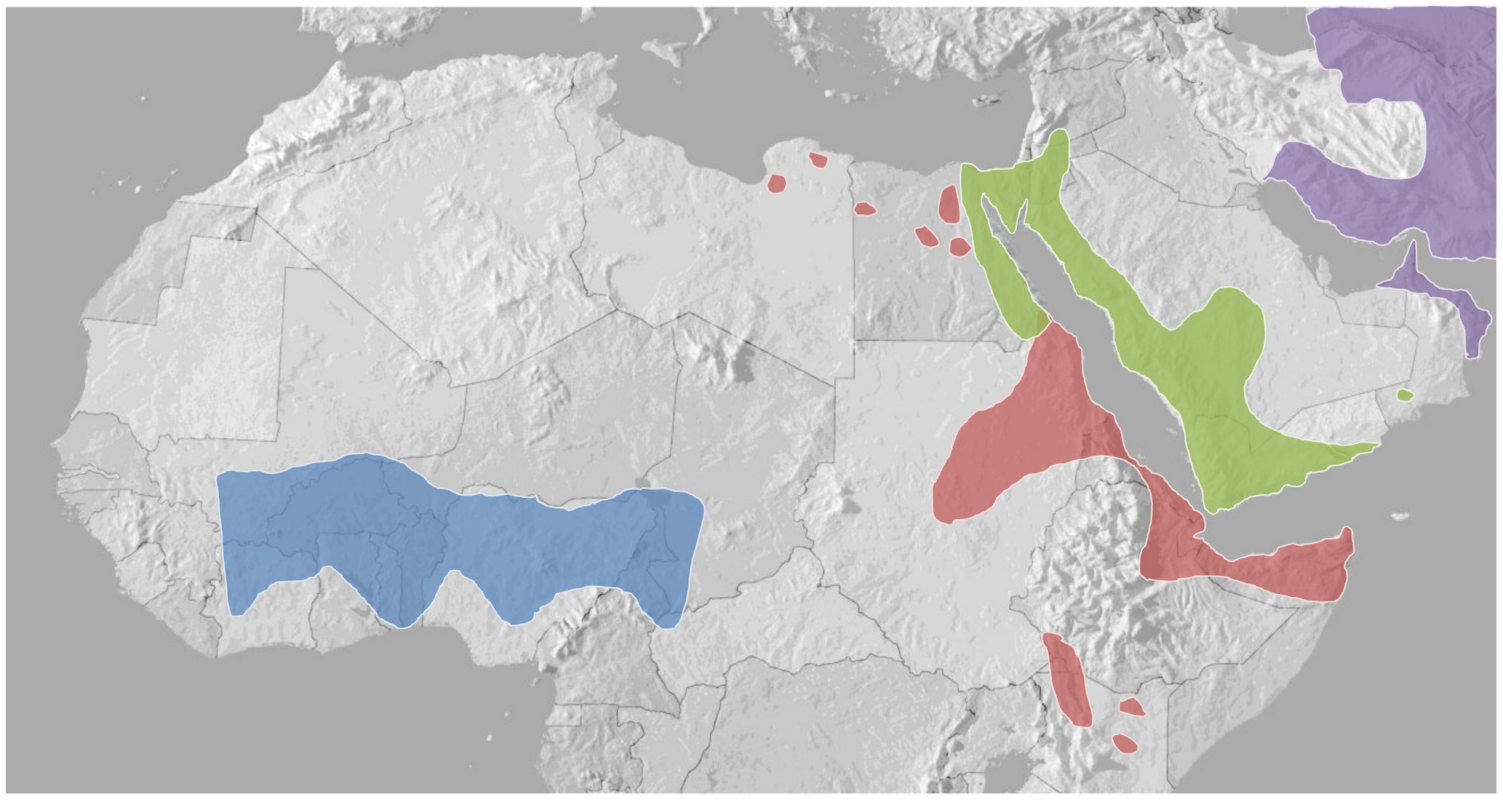
Distribution of four *Echis* species in Africa and the Middle East. Key: *E. ocellatus* – blue, *E. pyramidum* – red, *E. coloratus* – green, *E. carinatus* – purple. Distributions mapped according to the WHO venomous snake distribution database and a recent study of the genus *Echis* [16], [35].

The saw-scaled viper, *Echis ocellatus*, is responsible for most snakebite-deaths in Nigeria [10]–[12]; the other medically-important snakes are the puff adder, *Bitis arietans*, and the spitting cobra, *Naja nigricollis* [13], [14]. EchiTAb-Plus-ICP is a new equine polyspecific IgG antivenom developed in Costa Rica to treat envenoming by all three snake species [7]. In view of the very high number of *E. ocellatus* bites in Nigeria and the severe haemorrhaging and incoagulable bleeding experienced by systemically envenomed victims, an additional ovine IgG antivenom, EchiTAbG (MicroPharm Ltd) was manufactured in Wales for the treatment of *E. ocellatus* envenoming and has been registered (A6-0078) by the Nigerian medicines authority, NAFDAC. Since *E. ocellatus* is widely distributed across the West African savannah (Figure 1), EchiTAbG offers considerable therapeutic promise in many countries in the region. With the intent of maximising the clinical contribution of this new antivenom and cognisant that (i) other *Echis* species represent public health concerns in East (*E. pyramidum*) and North-East (*E. coloratus*) Africa (Figure 1) and (ii) that their venom protein family composition is not dissimilar to *E. ocellatus* [15], the objective of this study was to examine the pre-clinical intra-generic venom-neutralising efficacy of EchiTAbG.

## Materials and Methods

To achieve a more complete understanding of the immunology underpinning the cross-specific venom neutralising potential of EchiTAbG, we performed a series of assays to determine the IgG titre, specificity and relative avidity of sera raised in sheep immunised with venom from species representing each taxonomic group of the *Echis* genus in Africa [16] as follows: (i) *E. pyramidum leakeyi* (Kenya) representing the *pyramidum* complex which includes *E. leucogaster* and *E. p. pyramidum*, (ii) *E. coloratus* (Egypt) representing this species and *E. omanensis*, (iii) *E. ocellatus* representing this species and *E. jogeri*. To assess the geographic limitation of the exercise we included venom from a non-African saw-scaled viper (*E. carinatus sochureki*, United Arab Emirates) in all the experiments, including provision of species-specific antisera.

### Snakes and venom extraction

Snakes were maintained in the Herpetarium at the Liverpool School of Tropical Medicine. Venom was extracted from wild-caught specimens of *E. ocellatus* (Nigeria), *E. coloratus* (Egypt), *E. p. leakeyi* (Kenya) and *E. c. sochureki* (UAE) on several occasions, pooled, frozen, lyophilised and stored at 4°C prior to reconstitution in phosphate-buffered saline (PBS).

### Immunisation and antiserum production

Antisera were generated against venom from *E. p. leakeyi*, *E. coloratus* and *E. c. sochureki* using protocols identical to that used in the production of the *E. ocellatus*-specific antivenom, EchiTAbG. Six sheep (two per venom) were initially immunised with 0.5mg of venom emulsified with Freund's Complete Adjuvant followed by boosting immunisations of 1.0mg of venom emulsified with Freund's Incomplete Adjuvant every 28 days. To maximise seroconversion, venom immunisations were administered subcutaneously in six sites close to the major draining lymph nodes in the neck and groin. Blood samples were taken 14 days after immunisation.

Once the anti-venom IgG had reached a plateau (16 weeks, personal communication, MicroPharm Ltd) one litre of blood was taken, allowed to clot, centrifuged and the sera stored at −20°C. IgG was extracted by the addition of caprylic acid (Sigma, UK) to a final concentration of 5% and stirring vigorously for two hours to precipitate non-IgG proteins. The suspension was centrifuged at 13,000 rpm (in a microcentrifuge) for 60 min and the supernatant IgG dialysed with three changes of 20 mM sodium phosphate buffer (pH 7.4). All the IgG preparations were formulated to the same concentration, 30mg/ml in PBS, and stored at −20°C. IgG generated against *E. ocellatus* venom and the *E. ocellatus* antivenom EchiTAbG were obtained from MicroPharm Ltd.

Note: to avoid possible confusion between the antivenoms resulting from commercial manufacture and the anti-*Echis* species IgG antivenoms prepared in our laboratory – the latter have been termed IgG antisera.

### Immunological assays

#### a) End Point Titration (EPT) ELISA

Ninety six (96) well ELISA plates (Nunc) were coated with 100ng of venom (a separate plate for each *Echis* species) prepared in carbonate buffer, pH 9.6 and the plates incubated at 4°C overnight. Plates were washed after each stage, using 6 changes of TBST (0.01 M Tris-HCl, pH 8.5; 0.15 M NaCl; 1% Tween 20). Next, the plates were incubated at room temperature (RT) for 3 hours with 5% non-fat milk (diluted with TBST) to ‘block’ non-specific reactivity. The plates were then washed and incubated (in duplicate) with each of the four *Echis* species-specific IgG antisera, at an initial dilution of 1∶100 followed by 1∶5 serial dilutions and incubated overnight at 4°C. The plates were washed and then incubated in horseradish peroxidise-conjugated goat anti-sheep IgG (1∶1000; Sigma, UK) for 3 hours at RT. The results were visualized by addition of substrate (0.2% 2,2/-azino-bis (2-ethylbenzthiazoline-6-sulphonic acid) in citrate buffer, pH 4.0 containing 0.015% hydrogen peroxide (Sigma, UK) and optical density (OD) measured at 405nm. The titre is described as the dilution at which absorbance was greater than that of the negative control (IgG from non-immunised sheep) plus 2 standard deviations.

#### b) Relative avidity ELISA

This assay was performed as above except that the IgG antisera were diluted to a single concentration of 1∶10,000, incubated overnight at 4°C, washed with TBST and the chaotrope, ammonium thiocyanate, added to the wells in a range of concentrations (0–8 M) for 15 minutes. Plates were washed, and all subsequent steps were the same as the End Point Titration ELISA. Relative avidity was determined as the percentage reduction in ELISA OD reading (measured at 405nm) between the maximum (8 M) and minimum (0 M) concentrations of ammonium thiocyanate.

#### c) SDS-PAGE and immunoblotting

The lyophilised *Echis* species venoms were reconstituted to 1mg/ml in reduced protein loading buffer and boiled for ten minutes. Seven µg of venom, together with molecular weight marker (Broad range molecular weight protein markers, Promega) was added to a 15% SDS-PAGE gel and fractionated under 200 volts and the resultant proteins visualised by staining with Coomassie Blue R-250.

Immunoblotting was carried out in the same way except that after electrophoresis, the gels were electro-blotted onto 0.45µm nitrocellulose membranes using the manufacturer's protocols (Bio-Rad, UK). Following confirmation of successful protein transfer by reversible Ponceau S staining, the membranes were incubated overnight in blocking buffer (5% non-fat milk in TBST), followed by six washes of TBST over 90 minutes and incubation overnight with primary antibodies (the *E. ocellatus*-, *E. p. leakeyi*-, *E. coloratus*- and *E. c. sochureki*-specific IgG) diluted 1∶5,000 in blocking buffer. Blots were washed as above, then incubated for 2 hours with horseradish peroxidise-conjugated donkey anti-sheep secondary antibody (1∶2,000 dilution) before a final wash with TBST and visualisation after the addition of DAB substrate (50 mg 3,3-diaminobenzidine, 100 ml PBS and 0.024% hydrogen peroxide; Sigma, UK).

#### d) Small scale affinity purification

To assess the cross-reactivity of the four *Echis* species-specific IgG antisera to venoms under native conditions (and conditions distinct to that of the ELISA assays), we prepared small scale affinity columns for each of the venoms. 1g of CNBr-activated 4 Fast Flow Sepharose (GE Healthcare, UK) was swollen, washed with 1mM HCl, transferred to a 3.5ml column (Bio-Rad, UK) and washed twice with 0.1M sodium hydrogen carbonate pH 8.3. Five mg of venom (1mg/ml 0.1M sodium hydrogen carbonate pH 8.3 solution) was coupled with the Sepharose by end-over-end mixing at 4°C overnight. Columns were drained and active groups blocked by end-over-end mixing for 2 hours with 1M Ethanolamine-Cl pH 9.0, washed in washing buffer (0.1M sodium phosphate pH 7.5 containing 0.5M NaCl) and eluted (0.1M glycine pH 2.5 containing 0.1M HCl) before storage at 4°C. Columns were equilibrated at RT and washed in washing buffer before 3mg of the species-specific IgG antisera (1mg/ml in washing buffer) was added to the column and mixed overnight. Columns were subsequently washed and eluted. The eluate was concentrated using 5 kDa cut-off Vivaspin columns (Sartorius Stedim Biotech, UK) and quantified using a LD1000 series NanoDrop spectrophotometer (Thermo Scientific, USA). The results were calculated as a percent of the 3mg of species-specific IgG added to each column.

### Pre-clinical assays

All animal experimentation was conducted using protocols approved by the University of Liverpool Animal Welfare Committee and performed under licenced approval of the UK Home Office.

#### a) Venom lethality

As an essential prerequisite to assessing antivenom efficacy, we determined the median lethal dose (LD_50_) for each of the four *Echis* venoms in mice using WHO-recommended protocols [17] refined to reduce the duration of the procedure [18]. Briefly, groups of five male CD-1 mice (18–20g) received an intravenous (iv) tail injection of varying doses of venom in 100µl PBS and, 7 hours later, the number of surviving mice in each group was recorded. The venom LD_50_ (the amount of venom that kills 50% of the injected mice) and 95% confidence limits of each *Echis* species was calculated using probit analysis [19].

#### b) Antivenom neutralisation of venom lethality

To compare the effectiveness of EchiTAbG to neutralise the lethal effects of each of the *Echis* species venoms, we used the median effective dose (ED_50_) assay; a WHO-recommended test for determining the least amount of antivenom required to prevent death in 50% of mice injected with five venom LD_50_s. Using a previously described protocol [18], various doses of EchiTAbG antivenom were mixed with 5 venom LD_50_s and the final volume made up to 200µl with PBS and the mixture incubated at 37°C for 30 minutes. The mixture was iv injected into the tail vein of groups of 5 CD-1 mice and, 7 hours later, the number of surviving mice in each group was recorded. The median effective dose (ED_50_) and 95% confidence limits were calculated using probit analysis [19].

The 7 hour time frame for the LD_50_ and ED_50_ assays was used instead of the more conventional 24 hour period as a result of numerous earlier assays which revealed that over 98% mice succumbed to *E. ocellatus* envenoming within 7 hours of injecting the venom or venom/antivenom mixture (data not shown). These observations, with this venom, permitted us to make this humane reduction in the duration of the experiments without risking invalidating the results.

## Results

### Immunological profiles of the *Echis* species-specific IgG antisera

**Figure 2:**
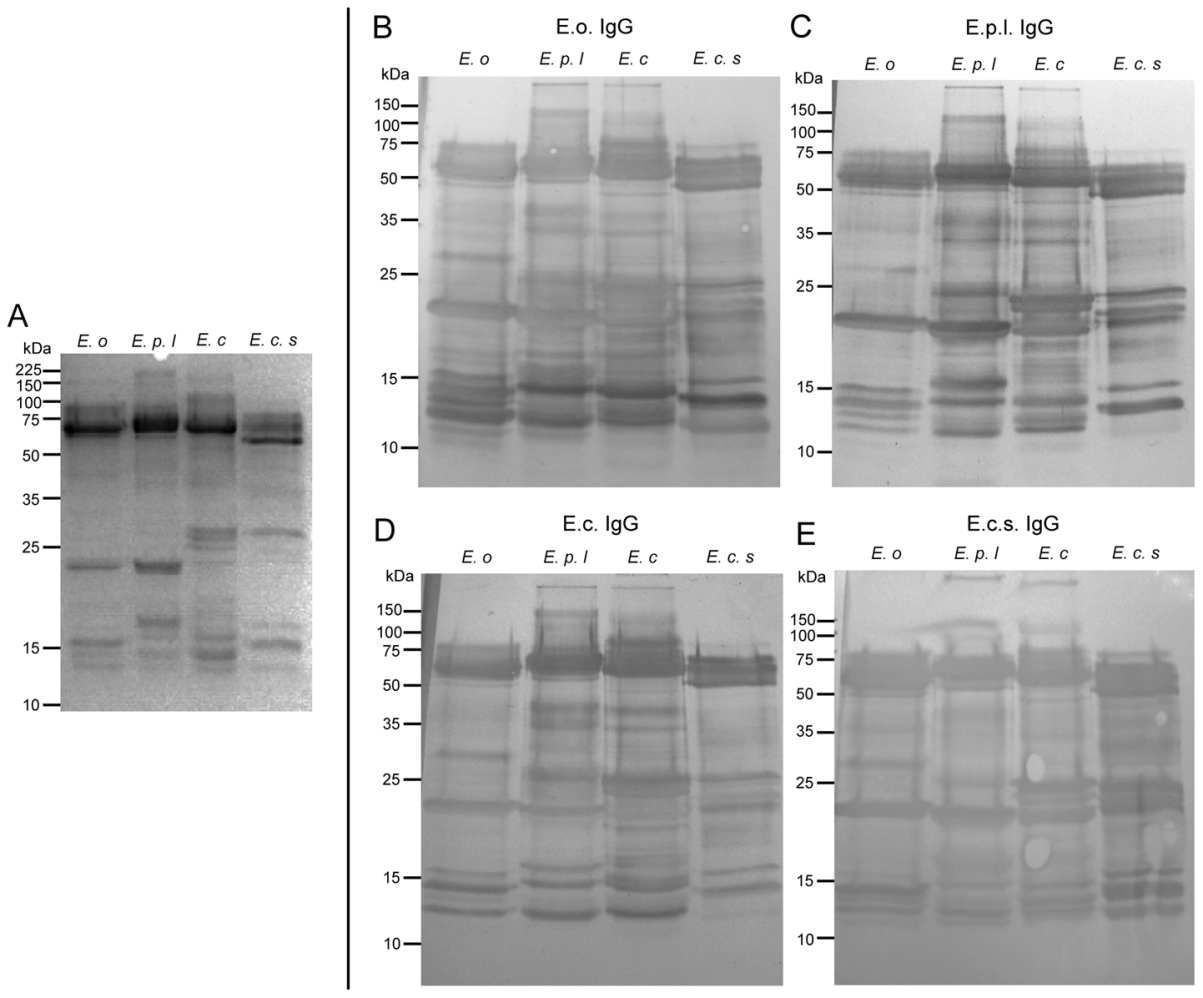
*Echis* species-specific IgG antisera exhibit extensive cross-specific venom protein reactivity. The venom proteins of *E. ocellatus (E.o)*, *E. p. leakeyi (E.p.l)*, *E. coloratus (E.c)* and *E. c. sochureki (E.c.s)* visualised using reduced SDS-PAGE (A), after immunoblotting, showed extensive cross-specific reactivity with IgG antisera specific to *E. ocellatus* (B), *E. p. leakeyi* (C), *E. coloratus* (D) *E. c. sochureki* (E). The sera were diluted 1∶5,000 and 7µg of each venom was used in all gels.

The reduced SDS-PAGE profiles reveal intra-generic variation in molecular mass and quantitative representation of the venom proteins present in the four *Echis* venoms (Figure 2A). However, immunoblotting of the four venoms with each of the four *Echis* species-specific IgG antisera demonstrated that the intensity of immunoreactivity of each IgG antiserum to proteins of the homologous venom was matched by that to the three heterologous venoms (Figure 2B–E). Indeed, such was the intensity of the cross-species IgG immunoreactivity that we were unable to detect any protein in the SDS-PAGE venom profiles that was not reactive with each of the four *Echis* species-specific IgG antisera. Furthermore, the immunoblots revealed the existence of more venom proteins than was apparent from the SDS-PAGE. We interpret this analysis as illustrating that while intra-generic differences in the size of the venom proteins exist, these proteins were likely to be size-variants of the same protein families.

**Figure 3:**
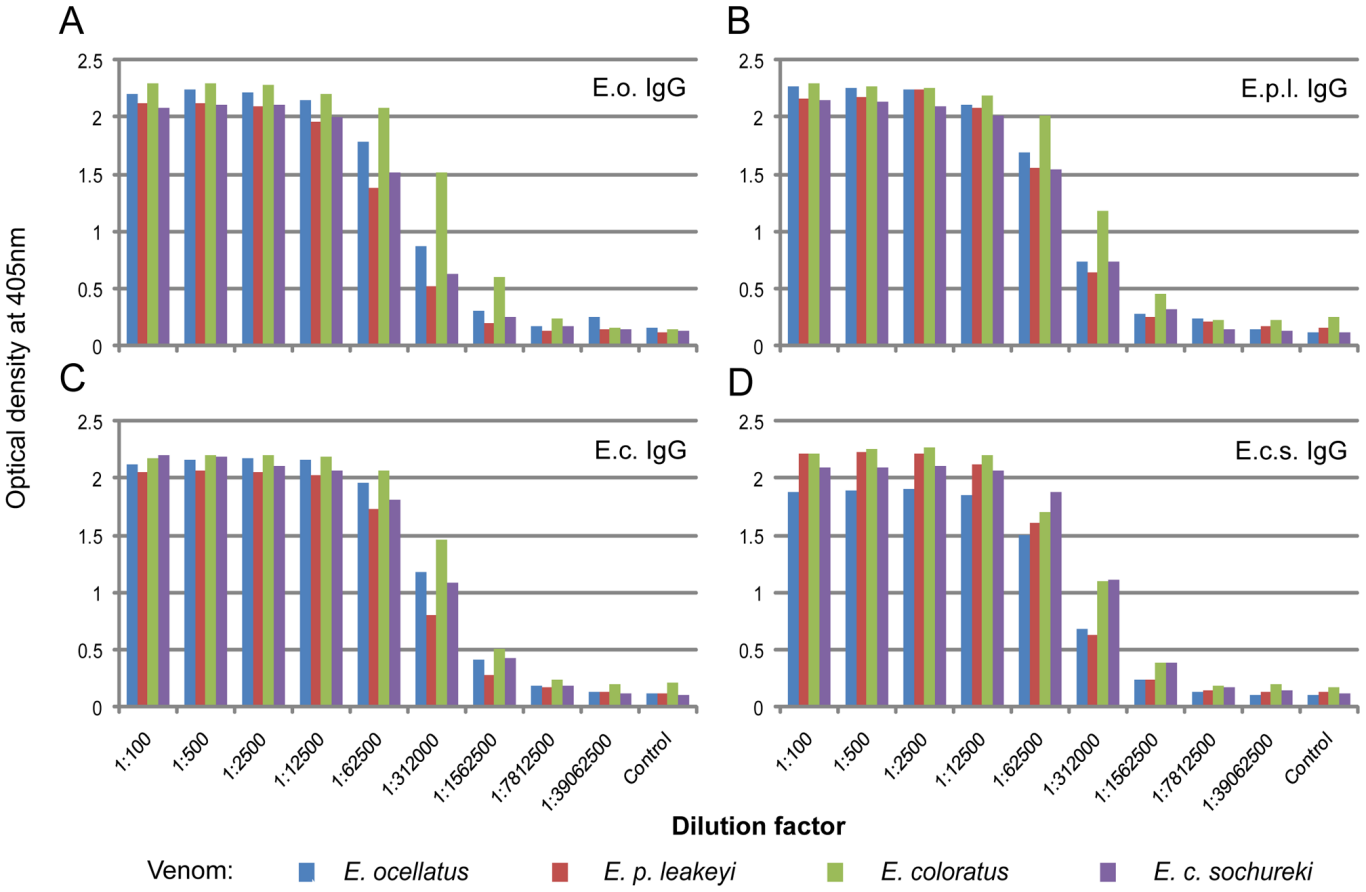
Extensive cross-specific immunological reactivity between *Echis* venoms and *Echis* species-specific IgG antisera revealed by End Point Titration ELISA. Venom from *E. ocellatus* (blue bars), *E. p. leakeyi* (red bars), *E. coloratus* (green bars) and *E. c. sochureki* (purple bars) were incubated with serial dilutions (horizontal axis) of IgG antisera raised against *E. ocellatus* (A), *E. p. leakeyi* (B), *E. coloratus* (C) and *E. c. sochureki* (D) and the optical density determined (vertical axis).

The immunogenicity of each *Echis* venom was assessed using the EPT ELISA assay to determine the IgG titre of each of the four *Echis* species-specific IgG antisera to each *Echis* venom (Figure 3A–D). The overall plateau and then decline of IgG titre after successive IgG dilution was strikingly similar for each of the IgG antisera; as reflected in the assigned EPT IgG titres (Table 1). The slightly slower decline of the *E. coloratus* venom-antisera profiles against each of the venoms (except the *E. c. sochureki* venom, Figure 3D) suggested that *E. coloratus* venom is perhaps more immunogenic than the other *Echis* venoms. Similarly, because the *E. p. leakeyi* IgG antisera showed the most rapid decline in IgG titre against its homologous and the heterologous venoms, it could be surmised that *E. p. leakeyi* venom is the least immunogenic. However, these differences were minor and we therefore caution against assigning much immunological significance to these observations.

**Table 1:** The End Point Titre of each *Echis* species-specific IgG antisera to each *Echis* venom.

	*Echis* species-specific IgG antisera			
Venom	*E. ocellatus*	*E. p. leakeyi*	*E. coloratus*	*E. c. sochureki*
***E. ocellatus***	*1.56×10^−06^*	3.12×10^−05^	1.56×10^−06^	1.56×10^−06^
***E. p. leakeyi***	1.56×10^−06^	*3.12×10^−05^*	1.56×10^−06^	1.56×10^−06^
***E. coloratus***	7.81×10^−06^	1.56×10^−06^	*1.56×10^−06^*	1.56×10^−06^
***E. c. sochureki***	1.56×10^−06^	1.56×10^−06^	1.56×10^−06^	*1.56×10^−06^*

Italicised values highlight homologous combinations of venom-IgG antisera.

Considerable intra-generic immunological cross-reactivity was noted from the immunoblotting and ELISA assay when the venoms were in ‘reduced’ or ‘native’ states (respectively). We next wished to examine this immunological cross-reactivity using a technology also using ‘native’ venoms but in a manner that would likely present the venom proteins to the IgG in a different configuration - and perhaps better reflecting the *in vivo* situation - than that achieved in the ELISA assay. We therefore prepared small scale CnBr-activated venom-affinity columns for each *Echis* venom and measured the amount of each *Echis* species-specific IgG that remained bound to the column after extensive washing of the column (Table 2). This new assay to measure venom-antivenom interactions revealed, in each case, that highest binding occurred between the homologous combination of venom and IgG antisera. This was the first assay to indicate that there are intra-generic differences in *Echis* venoms with immunological significance.

**Table 2:** Venom affinity-chromatography to measure the binding strength of each *Echis* species-specific antisera to each venom.

	*Echis* species-specific IgG antisera			
Venom Column	*E. ocellatus*	*E. p. leakeyi*	*E. coloratus*	*E. c. sochureki*
***E. ocellatus***	*10.23*	5.12	6.77	7.51
***E. p. leakeyi***	8.32	*8.02*	6.95	7.53
***E. coloratus***	8.44	7.71	*9.28*	9.38
***E. c. sochureki***	8.11	4.95	6.90	*11.12*

The amount of IgG from each *Echis* species specific antisera that remained bound to the *Echis* venom affinity chromatography column is displayed as a percent of the 3 mg of each IgG added to the column. Italicised values highlight homologous venom-antivenom results.

**Figure 4:**
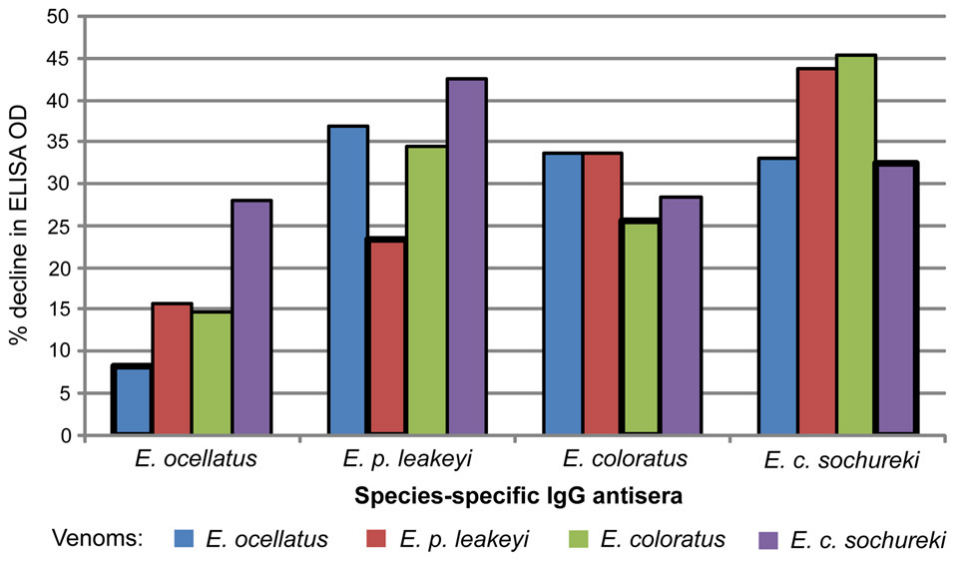
The Relative Avidity of each *Echis* species-specific IgG antisera to each *Echis* venom. Results are expressed as the percentage decline in ELISA optical density (405nm) from the control (incubation with 0 M ammonium thiocyanate) to the treatment (8 M). Boxed bars highlight homologous venom-antivenom results.

To determine the strength of venom-antivenom binding in the presence of ammonium thyiocyanate (which disrupts protein-protein interactions), we used the Relative Avidity ELISA assay to examine the titre of the four *Echis* species-specific IgG antisera to each of the venoms in the presence of increasing amounts of the chaotrope (Figure 4). Consistent with the results of the small scale venom affinity assay, the venom-antivenom interactions least affected by the chaotrope, therefore exhibiting the strongest binding, were between the homologous combinations of venom and IgG antisera. The *E. ocellatus* IgG antisera exhibited the strongest binding and the *E. c. sochureki* IgG antisera showed the overall weakest binding to other *Echis* venoms (Figure 4).

### Pre-clinical tests of venom lethality and venom-neutralising efficacy of EchiTAbG

The lethal effects (expressed as LD_50_s) of the four *Echis* venoms to mice, ranged from 9.81µg venom/mouse for *E. coloratus* to 15.10µg for *E. c. sochureki*. The 95% confidence limits indicate there is no significant difference between the venom lethalities of the four *Echis* species (Table 3).

**Table 3:** Venom lethality and venom-neutralising efficacy of (A) EchiTAbG and (B) the *E.c. sochureki* IgG antisera.

A)	LD_50_ (µg/mouse)	ED_50_ (µl/mouse)
Venom		EchiTAbG
***E. ocellatus***	12.43 (9.00–20.45)	58.46 (35.32–90.92)
***E. p. leakeyi***	13.55 (8.98–38.33)	64.87 (23.86–129.65)
***E. coloratus***	9.81 (6.06–19.25)	44.25 (21.90–58.29)
***E. c. sochureki***	15.10 (6.49–19.70)	NE

B)	LD_50_ (µg/mouse)	ED_50_ (µl/mouse)
Venom		*E. c. sochureki* IgG antisera
***E. c. sochureki***	15.10 (6.49–19.70)	54.42 (43.93–58.33)

The venom lethality for each *Echis* species is expressed as the Median Lethal Dose (LD_50_). The venom-neutralising efficacy (ED_50_) was determined for (A) EchiTAbG against venom from all the *Echis species* and (B) for the *E. c. sochureki* IgG antisera against *E. c. sochureki* venom. 95% confidence limits are displayed in parentheses. NE = Not effective.

Only slightly varying volumes of the *E. ocellatus* antivenom, EchiTAbG, was required to completely neutralise venom-induced lethality of the three African *Echis* species (*E. ocellatus*, *E. p. leakeyi* and *E. coloratus*; Table 3). Perhaps surprisingly in light of results of the small scale venom-affinity and Relative Avidity assays, EchiTAbG was more effective against *E. coloratus* venom than against its homologous venom, *E. ocellatus*. Importantly, EchiTAbG was ineffective at the maximal permitted volume of antivenom (100µl) against the Asian species, *E. c. sochureki*.

We were concerned with the apparently conflicting observations that although the *E. c. sochureki* venom exhibited the lowest toxicity (highest LD_50_ result), EchiTAbG was unable to neutralise its venom effects. We therefore performed an ED_50_ test with *E. c. sochureki* venom and its homologous IgG antisera (Table 3); the latter proving as effective against *E. c. sochureki* venom (54.42µl/mouse) as EchiTAbG against *E. ocellatus* venom.

The efficacy of EchiTAbG against the lethal effects of *E. ocellatus* venom noted in this study (an ED_50_ of 58µl antivenom/mouse: 1740µg antivenom/mouse) was substantially lower than that recently reported for this antivenom-venom combination (ED_50_ of 8µl) in a separate study [8]. The *E. ocellatus* venom LD_50_ results here and from the other study [8] are comparable and consistent with other publications [18], [20] indicating that the disparate ED_50_ results did not arise from batches of *E. ocellatus* venom with different toxicities. We have repeated the ED_50_ experiment with different batches of EchiTAbG antivenom and *E. ocellatus* venom in published [18] and unpublished experiments (data not shown) with results that confirm those obtained in this study.

The results of the ED_50_ assays demonstrate a lack of congruence between the results of *in vivo* pre-clinical tests and immunological assays. Notably, no single immunological assessment could be used to predict the pre-clinical efficacy of EchiTAbG. Only the Relative Avidity ELISA results indicated the potential ineffectiveness of EchiTAbG against *E. c. sochureki* venom (Figure 4). We interpret this as indicating that while all effective antivenoms require high levels of IgG titre, specificity and avidity [18], [21], these immunological characteristics cannot be used to predict antivenom efficacy.

## Discussion

Physicians throughout Africa are tasked with treating victims suffering life-threatening effects of envenoming that include systemic haemorrhage, coagulopathy, neurotoxicity and renal failure. Identifying the snake species is often difficult - making it problematic to select the most appropriate antivenom, which, in a resource-poor setting, are scarce and expensive. The problem is made more complex because the snake species responsible could be any of the following a) viper species (the *Echis* saw-scaled vipers; the puff adder, *B. arietans*; and several other *Bitis* species which, although incidences are rare can be potentially lethal), b) elapid species (the black-necked spitting cobra, *Naja nigricollis*; the Egyptian cobra, *N. haje*; the Mozambique spitting cobra, *N. mossambica*; the forest cobra, *N. melanoleuca*; species of the mamba genus including the black mamba, *Dendroaspis polylepis* and green mambas of East Africa, *D. angusticeps* and Central/West Africa, *D. jamesoni* and *D. viridis*) and c) the colubrid Boomslang, *Dispholidus typus*. Presumably in consideration of the above, the SAIMR polyspecific antivenom manufactured in South Africa (South African Vaccine Producer) includes venoms from many of the above snakes in its venom-immunisation mixture.

The EchiTAb Study Group's decision to manufacture an *E. ocellatus*-specific antivenom and a *E. ocellatus*, *B. arietans* and *N. nigricollis* polyspecific antivenom reflects the snakebite therapeutic needs of Nigeria. Epidemiological studies had identified these three species as being of greatest medical importance in the country [11]–[14], [22] and were the basis for deciding upon a polyspecific antivenom – EchiTAb-Plus-ICP produced in Costa Rica. The decision to manufacture an *E. ocellatus*-specific antivenom (EchiTAbG produced in UK) was based on (i) the unusually high *E. ocellatus*-bite incidence rate in Nigeria [11], (ii) the 10–20% fatality rate of untreated victims of *E. ocellatus* envenoming [10], [23] and (iii) that the 20 minute blood clotting test [24], [25] can be reliably used to distinguish *E. ocellatus* envenoming from other West African venomous species – facilitating the physicians antivenom-selection decision. Another consideration was that the dose-efficacy of monospecific antivenoms is typically greater than polyspecific antivenoms (the curative dose of EchiTAbG is one vial and three vials for EchiTAb-Plus-ICP) and thus monospecific antivenoms offer a more cost effective treatment option – providing that the snake species can be identified using either distinct symptomatology or species identification. The incidence of antivenom-induced adverse effects of both the EchiTAb Study Group antivenoms is low [8], perhaps because both are produced under sterile GMP conditions employing caprylic acid to select IgG from sera/plasma. EchiTAbG is now a registered medicine in Nigeria (A6-0078) and EchiTAb-Plus-ICP is currently in the process of registration.

In addition to providing the Nigerian Federal Ministry of Health with the monospecific EchiTAbG and polyspecific EchiTAb-Plus-ICP antivenoms, the EchiTAb Study Group has also provided ‘best-practice’ training of hospital physicians in (i) the clinical use of these antivenoms, (ii) treatment of adverse effects and (iii) surgical treatment of the tissue-necrotic effects of local envenoming. Training was also given in the use of snake-identification and symptomology of envenoming to assist in making the most cost-effective and clinically-effective antivenom-selection decisions. The EchiTAb Study Group also provided ambulances to improve the speed of antivenom treatment of envenomed victims in an effort to improve the clinical outcome. The EchiTAb Study Group considered this multi-faceted approach to snakebite treatment as the most effective means of addressing the variant needs of snakebite victims in the region.

EchiTAbG is being provided free to patients in two hospitals in Nigeria (Kaltungo, Gombe State and Zamko, Plateau) where admitting 30 snakebite victims a day is not unusual, particularly in the biannual rain seasons. While it is the intent of the EchiTAb Study Group to expand the geographical delivery of its antivenoms, the current scarcity of effective antivenom in the region has resulted in victims undertaking long and expensive journeys to attend these hospitals, with some victims reportedly travelling from as far as Cameroon in the East and Niger in the North-West (personal observation, AN and ND). These observations indicate the paucity of effective and affordable antivenom in West Africa where snakebite, and particularly *E. ocellatus*, is a medical problem in most countries (Burkina Faso [26], Mali [27] Côte d'-Ivoire [28], Ghana [29], Benin [30], Niger [31] and Cameroon [32]). EchiTAbG therefore offers a therapeutic benefit in many countries other than Nigeria for which it was designed.

Since the East and North-East African *Echis* vipers are also a public health concern, our objective was to determine, at the pre-clinical level, whether the efficacy of EchiTAbG against *E. ocellatus* could be extended to these other *Echis* species. Ideally, our pre-clinical assays would have been conducted on venoms from all the African *Echis* species but we could not justify the ethical and financial costs of such a large number of mice. Therefore, based upon the most comprehensive taxonomic study of the genus *Echis* [16], we selected a single species from each of the four distinct species complexes; (i) *E. p. leakeyi* (Kenya) as a representative of the *pyramidum* complex which also includes *E. leucogaster* and *E. p. pyramidum*, (ii) *E. coloratus* (Egypt) as a representative of this species and *E. omanensis*, (iii) *E. ocellatus* as a representative of this species and *E. jogeri* and (iv) *E. c. sochureki* (United Arab Emirates) as a representative of the Asiatic *carinatus* complex. Our earlier work on the venom gland transcriptomes of these representative *Echis* species revealed considerable intra-generic differences in the number of isoforms comprising the main *Echis* toxin groups (snake venom metalloproteinases, phospholipases A_2_, serine proteases, C-type lectins) [15]. However, each pathogenic toxin family was represented in all the *Echis* species [15] – a result suggesting the possibility that EchiTAbG would have cross-*Echis* species efficacy.

However, we were also aware of previous clinical failures of the ‘heterologous’ administration of *Echis* species-specific antivenoms [33], [34]. Consequently, and in line with WHO recommendations [17], we performed here a series of immunological assays examining the immunological venom cross reactivity of ovine IgG raised against each representative *Echis* species. The results of these assays, which were designed to measure IgG titre, specificity and relative avidity to venoms in reduced and native states, indicated a very considerable degree of immunological cross-reactivity of each species-specific IgG antisera to each *Echis* venom. However, EchiTAbG was ineffective in neutralising the *in vivo* lethal effects of *E. c. sochureki*. This indicates that while these immunological tests provide informative and comprehensive immunological profiles of an antivenom, they can not yet replace pre-clinical *in vivo* testing to indicate the efficacy of an antivenom.

The most important result of the study was that EchiTAbG neutralises the lethal effects of venom from East and North-East African *Echis* species (*E. p. leakeyi* and *E. coloratus*) with an efficacy equal to that it shows against *E. ocellatus* from West Africa. A recent study reports a similar potential for the other EchiTAb Study Group antivenom, EchiTAb-Plus-ICP [20]. While these pre-clinical results require verification in human clinical trials, they do indicate a wider than intended application for both EchiTAbG and EchiTAb-Plus-ICP. We believe this is vitally important to the sustained delivery of these new antivenoms, developed to resolve a crisis in antivenom supply to Nigeria, because their production is now vulnerable to the same fiscal insecurities that caused the antivenom crisis a decade ago. A greater market, through geographical expansion, should permit the application of economies of scale that hopefully will, sequentially, reduce costs to the purchasing ministries of health, increase demand and improve the delivery of these urgently needed life-saving therapies.
